# Supplementary material for: A Likelihood Approach for Real-Time Calibration of Stochastic Compartmental Epidemic Models
Source: PLoS Comput Biol. 2017 Jan 17;13(1):e1005257. doi: 10.1371/journal.pcbi.1005257 (PMC5240920; doi:10.1371/journal.pcbi.1005257)
Supplement: S1 File — (TAR.GZ) [file pcbi.1005257.s014.tar.gz › HSPH_Online-SI-Revision/EnKF07/n100-extreme/n100-extreme_EnKF2_table-8.pdf]

1	newly 3 weeks spec	1767.	1011.86	{267., 1774.}	59.1443	0.441818	newly 3 weeks cumul	3085.	20 080.9	{6844., 56 863.}	550.919	0.666044	newly 5 weeks spec	5804.	161.78	{5., 252.}	97.2126	2.1573	newly 5 weeks cumul	12 241.	20 599.3	{7035., 56 895.}	94.1403	0.222729	newly 62 weeks cumul	91 729.	20 886.1	{7868., 56 895.}	77.9307	0.784012
2	newly 3 weeks spec	2838.	1030.08	{160., 926.}	88.5595	0.827252	newly 3 weeks cumul	5925.	5566.48	{2306., 5168.}	74.534	0.295334	newly 5 weeks spec	5914.	506.59	{3., 590.}	94.7699	1.78527	newly 5 weeks cumul	16 050.	6793.39	{2741., 5266.}	82.2589	0.605284	newly 58 weeks cumul	82 551.	7891.43	{3005., 6711.}	91.3306	1.21194
3	newly 3 weeks spec	1557.	2596.44	{3., 7314.}	175.231	1.29626	newly 3 weeks cumul	2607.	34 624.6	{7198., 74 358.}	1232.05	0.995359	newly 5 weeks spec	6571.	987.88	{0., 3177.}	86.756	2.65862	newly 5 weeks cumul	12 454.	37 152.1	{7438., 74 358.}	215.675	0.467439	newly 63 weeks cumul	90 095.	39 425.6	{7479., 74 358.}	60.8929	0.521575
4	newly 3 weeks spec	302.	9147.07	{365., 17 723.}	2936.9	1.26144	newly 3 weeks cumul	421.	46 853.9	{10 261., 91 853.}	11 030.6	1.9016	newly 5 weeks spec	3492.	4861.24	{4., 10 537.}	110.227	0.973402	newly 5 weeks cumul	5026.	58 485.	{12 645., 96 657.}	1070.02	0.993681	newly 67 weeks cumul	90 300.	69 946.1	{14 195., 105 420.}	31.1557	0.262692
5	newly 3 weeks spec	841.	7095.14	{902., 20 671.}	748.982	0.755922	newly 3 weeks cumul	1316.	44 379.4	{10 958., 92 402.}	3272.3	1.37224	newly 5 weeks spec	3644.	1829.63	{96., 4025.}	62.6726	0.666197	newly 5 weeks cumul	6776.	49 723.6	{12 772., 104 851.}	639.073	0.750754	newly 64 weeks cumul	88 461.	52 451.1	{14 361., 107 204.}	52.6854	0.408742
6	newly 3 weeks spec	1468.	1033.7	{274., 1729.}	56.0668	0.351924	newly 3 weeks cumul	2440.	20 801.9	{8185., 52 159.}	752.539	0.796335	newly 5 weeks spec	5118.	157.3	{6., 240.}	96.9265	2.07077	newly 5 weeks cumul	10 351.	21 305.7	{9032., 54 966.}	114.829	0.239608	newly 62 weeks cumul	91 404.	21 754.	{9070., 54 966.}	76.2001	0.744927
7	newly 3 weeks spec	1624.	1825.08	{0., 923.}	168.935	1.55468	newly 3 weeks cumul	2704.	6334.27	{642., 12 074.}	205.988	0.421216	newly 5 weeks spec	5875.	740.45	{0., 299.}	95.2209	2.65883	newly 5 weeks cumul	11 979.	8418.59	{743., 20 699.}	115.386	0.835729	newly 60 weeks cumul	90 884.	9299.86	{837., 27 394.}	90.0563	1.55483
8	newly 3 weeks spec	1359.	938.19	{251., 1764.}	61.6446	0.413359	newly 3 weeks cumul	2461.	21 201.1	{4955., 63 036.}	763.291	0.760454	newly 5 weeks spec	5170.	167.63	{3., 253.}	96.7576	2.19319	newly 5 weeks cumul	10 407.	21 683.7	{6927., 64 463.}	126.54	0.266391	newly 62 weeks cumul	92 361.	22 530.7	{6984., 64 591.}	76.2965	0.775092
9	newly 3 weeks spec	953.	1520.21	{382., 1847.}	107.749	0.306223	newly 3 weeks cumul	1486.	27 790.6	{8729., 68 306.}	1771.1	1.08588	newly 5 weeks spec	4055.	239.32	{11., 265.}	95.015	1.81319	newly 5 weeks cumul	7477.	28 579.	{8881., 68 718.}	285.933	0.425052	newly 62 weeks cumul	92 411.	28 998.1	{9020., 68 728.}	71.3458	0.692619
10	newly 3 weeks spec	629.	9566.95	{1469., 21 005.}	1429.64	1.07622	newly 3 weeks cumul	952.	45 456.9	{13 055., 95 020.}	4677.64	1.54405	newly 5 weeks spec	4393.	4920.74	{47., 9865.}	71.8548	0.601779	newly 5 weeks cumul	6990.	57 206.7	{20 394., 108 999.}	725.343	0.863101	newly 66 weeks cumul	75 317.	72 481.7	{26 402., 111 906.}	40.8667	0.246081
11	newly 3 weeks spec	2514.	2295.75	{0., 4917.}	148.353	1.73325	newly 3 weeks cumul	4845.	8743.49	{947., 41 841.}	179.687	0.557212	newly 5 weeks spec	6044.	996.42	{0., 4321.}	92.4361	2.66112	newly 5 weeks cumul	15 023.	11 420.1	{947., 56 340.}	115.402	0.857072	newly 60 weeks cumul	84 746.	12 963.9	{947., 62 672.}	84.7395	1.40971
12	newly 3 weeks spec	1011.	214.55	{0., 541.}	81.4352	1.5137	newly 3 weeks cumul	1634.	5692.	{960., 12 218.}	267.685	0.453518	newly 5 weeks spec	4508.	52.37	{0., 86.}	98.8383	2.95624	newly 5 weeks cumul	8400.	5836.08	{1000., 12 893.}	63.2964	0.437796	newly 61 weeks cumul	92 398.	5983.39	{1005., 14 795.}	93.5243	1.38654
13	newly 3 weeks spec	2062.	2822.79	{158., 14 486.}	171.689	0.828244	newly 3 weeks cumul	3828.	13 009.8	{1319., 66 210.}	305.981	0.434036	newly 5 weeks spec	4926.	753.39	{14., 2170.}	89.5268	1.58417	newly 5 weeks cumul	12 079.	15 218.3	{1471., 74 350.}	151.032	0.691494	newly 59 weeks cumul	90 371.	16 817.1	{1524., 75 461.}	84.7069	1.26827
14	newly 3 weeks spec	207.	6532.59	{1069., 15 338.}	3057.31	1.32546	newly 3 weeks cumul	282.	40 125.7	{11 076., 92 223.}	14 129.7	2.00166	newly 5 weeks spec	1855.	1772.12	{104., 3626.}	76.7461	0.498245	newly 5 weeks cumul	2833.	45 204.2	{12 879., 104 809.}	1497.45	1.0788	newly 64 weeks cumul	71 218.	47 895.6	{14 222., 107 131.}	55.8267	0.386887
15	newly 3 weeks spec	874.	7208.16	{1074., 20 540.}	729.128	0.757379	newly 3 weeks cumul	1434.	45 213.1	{10 962., 95 999.}	3052.93	1.33842	newly 5 weeks spec	4922.	1958.04	{102., 4104.}	67.5973	0.724422	newly 5 weeks cumul	8480.	50 808.4	{12 628., 108 131.}	506.008	0.672143	newly 64 weeks cumul	79 509.	53 849.2	{14 199., 112 238.}	54.2427	0.385854
16	newly 3 weeks spec	582.	6352.25	{1145., 15 396.}	993.294	0.874806	newly 3 weeks cumul	805.	39 543.5	{10 590., 92 230.}	4813.78	1.54425	newly 5 weeks spec	4823.	1731.69	{98., 3539.}	69.5561	0.738554	newly 5 weeks cumul	7337.	44 496.8	{12 717., 104 864.}	510.722	0.675072	newly 64 weeks cumul	91 266.	47 165.2	{13 434., 107 186.}	57.7215	0.45883
17	newly 3 weeks spec	2320.	2520.29	{131., 14 542.}	154.475	0.920408	newly 3 weeks cumul	4411.	11 727.2	{1330., 51 750.}	247.415	0.454731	newly 5 weeks spec	5419.	748.09	{10., 2260.}	90.2932	1.7494	newly 5 weeks cumul	13 371.	13 823.6	{1487., 64 380.}	136.74	0.729446	newly 59 weeks cumul	87 876.	15 744.3	{1555., 69 651.}	85.6545	1.29284
18	newly 3 weeks spec	1347.	1786.95	{0., 1731.}	176.785	1.40703	newly 3 weeks cumul	2379.	7205.41	{934., 20 148.}	264.813	0.426872	newly 5 weeks spec	4153.	749.99	{0., 942.}	97.2687	2.43729	newly 5 weeks cumul	9012.	9247.77	{934., 27 462.}	133.072	0.738864	newly 60 weeks cumul	79 943.	10 490.	{934., 34 663.}	87.6329	1.45661
19	newly 3 weeks spec	927.	1153.07	{293., 1772.}	77.9752	0.293668	newly 3 weeks cumul	1494.	24 566.7	{7196., 68 280.}	1546.63	1.03311	newly 5 weeks spec	4254.	185.39	{9., 241.}	95.642	1.91997	newly 5 weeks cumul	7918.	25 145.9	{8771., 68 640.}	224.702	0.379394	newly 62 weeks cumul	93 229.	25 636.1	{9031., 68 652.}	73.8908	0.742708
20	newly 3 weeks spec	53.	9166.89	{376., 18 615.}	17 197.1	1.97196	newly 3 weeks cumul	74.	47 800.5	{10 982., 95 992.}	64 495.3	2.69161	newly 5 weeks spec	1059.	4875.98	{2., 11 491.}	411.764	1.12102	newly 5 weeks cumul	1395.	59 476.4	{14 152., 96 624.}	4164.01	1.53587	newly 67 weeks cumul	91 568.	70 867.6	{14 220., 105 263.}	30.4385	0.226515
21	newly 3 weeks spec	845.	6318.3	{1053., 15 249.}	652.128	0.71666	newly 3 weeks cumul	1345.	41 052.5	{9478., 92 450.}	2952.23	1.32217	newly 5 weeks spec	4282.	1773.29	{98., 3978.}	67.153	0.727396	newly 5 weeks cumul	7677.	46 065.2	{12 794., 104 971.}	506.918	0.66994	newly 64 weeks cumul	83 732.	48 976.6	{13 586., 107 130.}	54.4915	0.420189
22	newly 3 weeks spec	804.	6853.02	{311., 15 992.}	773.736	0.789226	newly 3 weeks cumul	1237.	51 309.1	{17 262., 94 953.}	4047.86	1.51151	newly 5 weeks spec	4322.	2032.3	{3., 4170.}	78.341	1.17211	newly 5 weeks cumul	7480.	56 989.6	{17 478., 106 931.}	664.695	0.799669	newly 65 weeks cumul	84 795.	60 063.1	{17 736., 115 791.}	45.8652	0.314085
23	newly 3 weeks spec	2606.	1133.73	{76., 1918.}	90.8246	0.818772	newly 3 weeks cumul	5006.	6465.6	{2316., 6846.}	88.9664	0.250081	newly 5 weeks spec	5905.	470.66	{3., 805.}	92.9951	1.79866	newly 5 weeks cumul	15 055.	7664.77	{2752., 9676.}	84.3831	0.576845	newly 58 weeks cumul	84 036.	8613.14	{3018., 12 688.}	90.6087	1.20386
24	newly 3 weeks spec	1803.	739.59	{35., 928.}	79.8253	0.735544	newly 3 weeks cumul	3172.	4400.22	{2305., 5326.}	55.4294	0.160049	newly 5 weeks spec	5220.	376.12	{0., 615.}	92.9307	1.93537	newly 5 weeks cumul	11 528.	5300.48	{2757., 5994.}	67.5463	0.475452	newly 58 weeks cumul	92 404.	6343.96	{3012., 6951.}	93.1345	1.29166
25	newly 3 weeks spec	784.	241.28	{0., 520.}	78.324	1.2592	newly 3 weeks cumul	1188.	5058.27	{836., 6942.}	335.216	0.473054	newly 5 weeks spec	4655.	62.02	{0., 90.}	98.6677	2.80932	newly 5 weeks cumul	7743.	5231.64	{916., 8682.}	63.9881	0.437867	newly 61 weeks cumul	89 551.	5415.04	{953., 9089.}	93.9531	1.4235
26	newly 3 weeks spec	2337.	2364.71	{129., 13 405.}	148.38	0.923097	newly 3 weeks cumul	4561.	11 237.7	{1329., 50 921.}	231.321	0.45604	newly 5 weeks spec	5505.	711.64	{10., 2148.}	90.9817	1.77043	newly 5 weeks cumul	13 758.	13 216.2	{1481., 63 690.}	131.856	0.738141	newly 59 weeks cumul	89 914.	15 111.2	{1544., 69 243.}	86.546	1.31258
27	newly 3 weeks spec	3178.	2866.97	{0., 9373.}	149.319	1.79551	newly 3 weeks cumul	6240.	9103.28	{930., 45 344.}	159.262	0.641213	newly 5 weeks spec	6900.	1201.56	{0., 7888.}	90.44	2.67296	newly 5 weeks cumul	18 218.	12 460.1	{930., 63 743.}	109.801	0.917382	newly 60 weeks cumul	90 939.	13 984.6	{940., 65 616.}	84.8897	1.43328
28	newly 3 weeks spec	1642.	6248.17	{1176., 11 711.}	290.964	0.483052	newly 3 weeks cumul	2833.	41 648.4	{11 254., 94 539.}	1370.12	1.02094	newly 5 weeks spec	6075.	1850.38	{126., 3577.}	75.1806	0.830723	newly 5 weeks cumul	12 201.	46 774.2	{14 011., 108 033.}	288.055	0.479856	newly 64 weeks cumul	93 780.	49 914.5	{14 711., 112 095.}	55.4957	0.426954
29	newly 3 weeks spec	2186.	1142.88	{100., 1855.}	92.6029	0.750155	newly 3 weeks cumul	4260.	6457.83	{2306., 6699.}	97.3688	0.221294	newly 5 weeks spec	5160.	480.73	{2., 802.}	92.0905	1.76494	newly 5 weeks cumul	12 858.	7676.25	{2759., 9275.}	85.2637	0.523063	newly 58 weeks cumul	82 934.	8637.07	{3010., 12 306.}	90.4923	1.19767
30	newly 3 weeks spec	404.	9752.81	{377., 19 956.}	2323.99	1.18188	newly 3 weeks cumul	607.	46 831.4	{10 994., 89 118.}	7616.81	1.75489	newly 5 weeks spec	3670.	5189.98	{3., 12 451.}	109.243	0.967319	newly 5 weeks cumul	5636.	59 297.6	{13 240., 96 711.}	959.047	0.960526	newly 67 weeks cumul	93 251.	70 968.7	{14 152., 107 256.}	31.0419	0.262822
31	newly 3 weeks spec	169.	4853.53	{283., 10 956.}	2782.64	1.22151	newly 3 weeks cumul	246.	37 607.1	{4917., 90 856.}	15 187.4	1.93707	newly 5 weeks spec	2181.	3362.74	{1., 11 824.}	166.593	1.36041	newly 5 weeks cumul	3075.	44 792.8	{5797., 91 099.}	1359.89	0.985733	newly 68 weeks cumul	88 759.	57 210.9	{7042., 98 128.}	40.549	0.392168
32	newly 3 weeks spec	874.	6335.4	{306., 11 273.}	646.593	0.765009	newly 3 weeks cumul	1385.	60 783.9	{24 933., 110 638.}	4291.12	1.53269	newly 5 weeks spec	4137.	1536.81	{2., 3740.}	71.5526	1.13584	newly 5 weeks cumul	7503.	65 343.9	{27 434., 119 185.}	776.613	0.882478	newly 65 weeks cumul	77 539.	68 296.7	{33 004., 123 892.}	40.9833	0.241138
33	newly 3 weeks spec	1347.	2242.61	{2., 7299.}	183.105	1.4062	newly 3 weeks cumul	2306.	36 832.3	{7063., 76 718.}	1504.93	1.07198	newly 5 weeks spec	4307.	750.53	{0., 2961.}	84.8607	2.78236	newly 5 weeks cumul	9125.	38 798.2	{7312., 76 723.}	341.445	0.605923	newly 63 weeks cumul	92 452.	40 728.8	{7344., 76 723.}	60.5136	0.561773
34	newly 3 weeks spec	2211.	1080.4	{85., 979.}	91.1063	0.76274	newly 3 weeks cumul	4652.	5757.	{2288., 5247.}	77.9609	0.222957	newly 5 weeks spec	4361.	526.64	{1., 618.}	93.7744	1.75228	newly 5 weeks cumul	12 123.	7042.37	{2733., 5805.}	82.0613	0.502174	newly 58 weeks cumul	74 850.	8147.5	{2997., 6893.}	90.4089	1.16824
35	newly 3 weeks spec	845.	2832.05	{2., 8367.}	330.221	1.23627	newly 3 weeks cumul	1377.	43 969.4	{7261., 117 535.}	3093.13	1.3263	newly 5 weeks spec	3852.	824.82	{0., 3071.}	85.689	2.53071	newly 5 weeks cumul	7034.	46 224.6	{7494., 120 276.}	559.348	0.661921	newly 63 weeks cumul	93 735.	48 184.2	{7531., 120 462.}	59.6002	0.490937
36	newly 3 weeks spec	2415.	361.17	{0., 556.}	93.9101	1.58501	newly 3 weeks cumul	4659.	6449.71	{821., 18 379.}	101.198	0.388168	newly 5 weeks spec	5307.	88.78	{0., 92.}	98.3271	2.94465	newly 5 weeks cumul	13 623.	6709.3	{885., 18 388.}	73.204	0.624155	newly 61 weeks cumul	83 417.	6825.26	{904., 18 388.}	91.8179	1.34513
37	newly 3 weeks spec	569.	9213.34	{743., 20 761.}	1531.15	1.10638	newly 3 weeks cumul	794.	49 149.4	{13 055., 96 912.}	6091.62	1.65491	newly 5 weeks spec	3995.	4542.98	{21., 9638.}	72.4886	0.641551	newly 5 weeks cumul	6432.	60 177.6	{21 240., 111 747.}	840.426	0.907993	newly 66 weeks cumul	88 408.	73 021.	{31 961., 118 902.}	36.8411	0.220172
38	newly 3 weeks spec	174.	9146.82	{725., 20 908.}	5158.3	1.54556	newly 3 weeks cumul	245.	51 163.3	{16 006., 109 068.}	20 783.	2.20573	newly 5 weeks spec	2064.	4252.67	{7., 9136.}	145.966	0.733683	newly 5 weeks cumul	2893.	61 639.2	{22 618., 111 713.}	2031.6	1.25166	newly 66 weeks cumul	81 546.	72 897.9	{32 021., 124 017.}	38.9612	0.201261
39	newly 3 weeks spec	1689.	2441.08	{0., 4405.}	188.329	1.50545	newly 3 weeks cumul	3181.	8460.43	{939., 21 948.}	235.577	0.448358	newly 5 weeks spec	4543.	974.18	{0., 3150.}	96.405	2.41128	newly 5 weeks cumul	10 645.	11 203.4	{940., 27 330.}	133.329	0.740448	newly 60 weeks cumul	70 294.	12 462.2	{940., 35 114.}	86.9194	1.33231
40	newly 3 weeks spec	2733.	430.5	{0., 543.}	94.4201	1.58209	newly 3 weeks cumul	5234.	7267.22	{836., 18 412.}	107.356	0.414766	newly 5 weeks spec	6145.	131.35	{0., 97.}	97.8625	2.95022	newly 5 weeks cumul	15 669.	7625.61	{923., 18 469.}	74.9035	0.658873	newly 61 weeks cumul	84 270.	7829.16	{954., 18 469.}	90.7094	1.32086
41	newly 3 weeks spec	2085.	1176.18	{265., 1790.}	68.895	0.511515	newly 3 weeks cumul	3973.	20 046.7	{8715., 66 593.}	406.001	0.550408	newly 5 weeks spec	5334.	187.49	{7., 266.}	96.485	2.03365	newly 5 weeks cumul	12 888.	20 650.4	{8896., 68 498.}	92.1861	0.227725	newly 62 weeks cumul	72 211.	20 970.6	{8908., 68 510.}	73.3413	0.698535
42	newly 3 weeks spec	641.	10 038.2	{439., 21 140.}	1478.51	1.07295	newly 3 weeks cumul	992.	47 295.6	{12 840., 95 063.}	4671.51	1.52889	newly 5 weeks spec	4341.	5212.83	{92., 10 950.}	77.5132	0.644524	newly 5 weeks cumul	7191.	59 750.8	{17 611., 111 774.}	739.152	0.862722	newly 66 weeks cumul	73 182.	76 169.6	{28 100., 119 049.}	43.6289	0.230673
43	newly 3 weeks spec	1358.	1909.56	{147., 5670.}	162.728	0.657944	newly 3 weeks cumul	2359.	8595.11	{1332., 22 128.}	291.776	0.299215	newly 5 weeks spec	4809.	567.49	{11., 2043.}	92.4365	1.61657	newly 5 weeks cumul	9757.	10 166.1	{1482., 39 835.}	125.141	0.592905	newly 59 weeks cumul	92 457.	11 432.7	{1542., 48 236.}	87.6346	1.33929
44	newly 3 weeks spec	944.	6060.77	{269., 12 730.}	578.293	0.757263	newly 3 weeks cumul	1464.	56 296.5	{18 489., 102 263.}	3749.23	1.478	newly 5 weeks spec	6185.	1715.9	{1., 4206.}	78.5681	1.54912	newly 5 weeks cumul	10 162.	61 186.7	{18 593., 114 890.}	512.045	0.755644	newly 65 weeks cumul	91 937.	63 695.5	{18 593., 116 633.}	43.015	0.355974
45	newly 3 weeks spec	599.	6302.42	{1049., 15 341.}	954.781	0.857829	newly 3 weeks cumul	910.	40 384.9	{9484., 92 366.}	4339.48	1.4932	newly 5 weeks spec	4808.	1690.34	{99., 3581.}	70.3573	0.76566	newly 5 weeks cumul	7429.	45 248.7	{12 705., 104 705.}	515.158	0.684262	newly 64 weeks cumul	82 950.	47 819.6	{13 422., 107 087.}	56.0577	0.427794
46	newly 3 weeks spec	564.	6702.35	{950., 20 383.}	1092.37	0.88706	newly 3 weeks cumul	898.	39 890.9	{9457., 92 238.}	4343.85	1.47913	newly 5 weeks spec	3017.	1842.34	{89., 3650.}	62.4256	0.608685	newly 5 weeks cumul	5294.	45 175.	{12 715., 104 838.}	758.935	0.80791	newly 64 weeks cumul	85 120.	48 065.6	{13 260., 107 136.}	56.3134	0.45195
47	newly 3 weeks spec	765.	9156.65	{834., 21 266.}	1111.01	0.996489	newly 3 weeks cumul	1158.	44 489.8	{13 095., 91 269.}	3744.9	1.44143	newly 5 weeks spec	3839.	4782.86	{42., 9893.}	82.8752	0.652333	newly 5 weeks cumul	6738.	55 858.8	{19 270., 105 859.}	737.524	0.870049	newly 66 weeks cumul	82 631.	70 145.8	{23 371., 111 984.}	39.8726	0.281716
48	newly 3 weeks spec	268.	9195.42	{497., 20 752.}	3334.65	1.35765	newly 3 weeks cumul	370.	49 819.1	{13 126., 108 884.}	13 364.6	1.98054	newly 5 weeks spec	2437.	4513.22	{10., 9643.}	127.471	0.704184	newly 5 weeks cumul	3631.	60 768.5	{21 219., 112 397.}	1577.53	1.14215	newly 66 weeks cumul	91 693.	73 573.6	{30 833., 124 004.}	38.3591	0.23409
49	newly 3 weeks spec	151.	4782.14	{257., 10 886.}	3075.7	1.23484	newly 3 weeks cumul	195.	37 778.5	{4873., 94 405.}	19 273.6	2.02472	newly 5 weeks spec	3032.	3348.53	{0., 10 487.}	129.103	1.46848	newly 5 weeks cumul	3868.	44 914.	{5792., 96 709.}	1066.99	0.896931	newly 68 weeks cumul	88 820.	57 586.	{6541., 97 952.}	40.3681	0.394514
50	newly 3 weeks spec	2772.	2356.78	{162., 14 327.}	135.222	0.951352	newly 3 weeks cumul	5085.	10 885.3	{1334., 51 607.}	206.392	0.485837	newly 5 weeks spec	6948.	630.39	{14., 2167.}	91.7373	1.81226	newly 5 weeks cumul	16 731.	12 732.8	{1466., 64 649.}	119.113	0.807516	newly 59 weeks cumul	91 561.	14 169.4	{1522., 69 341.}	86.1236	1.32254
